# Quantification of circadian rhythms in mammalian lung tissue snapshot data

**DOI:** 10.1038/s41598-024-66694-7

**Published:** 2024-07-14

**Authors:** Saskia Grabe, Bharath Ananthasubramaniam, Hanspeter Herzel

**Affiliations:** 1 Charité Center for Basic Sciences, Institute for Theoretical Biology, Charité—Universitätsmedizin Berlin, Berlin, Germany; 2 Department of Biology, Institute for Theoretical Biology, Humboldt-Universität zu Berlin, Berlin, Germany

**Keywords:** Computational biology and bioinformatics, Systems biology

## Abstract

Healthy mammalian cells have a circadian clock, a gene regulatory network that allows them to schedule their physiological processes to optimal times of the day. When healthy cells turn into cancer cells, the circadian clock often becomes cancer specifically disturbed, so there is an interest in the extraction of circadian features from gene expression data of cancer. This is challenging, as clinical gene expression samples of cancer are snapshot-like and the circadian clock is best examined using gene expression time series. In this study, we obtained lists of intersecting circadian genes in public gene expression time series data of lung tissue of mouse and baboon. We base our circadian gene lists on correlations of gene expression levels of circadian genes, which are closely associated to the phase differences between them. Combining circadian gene expression patterns of diurnal and nocturnal species of different ages provides circadian genes that are also important in healthy and cancerous human lung tissue. We tested the quality of the representation of the circadian clock in our gene lists by PCA-based reconstructions of the circadian times of the mouse and baboon samples. Then we assigned potential circadian times to the human lung tissue samples and find an intact circadian clock in the healthy human lung tissue, but an altered, weak clock in the adjacent cancerous lung tissue.

## Introduction

Almost all mammalian cell types track the time of the day with a molecular clock, the so-called circadian clock, and coordinate intracellular processes at optimal times of the day. The gene regulatory framework of the circadian clock are transcriptional-translational-feedback-loops of clock genes^1^. Knocking-out clock genes experimentally severely disturbs the circadian clock^2^. All clock genes have functionally redundant paralogs and, except for *ARNTL*, multiple paralogs have to be knocked out together to disrupt the circadian clock^3^.

The most prominent transcriptional-translational-feedback-loop of the circadian clock starts with the transcription and translation of *ARNTL* and *CLOCK*, which assemble into a gene regulatory protein complex. This ARNTL:CLOCK complex activates the transcription of *PER* and *CRY*, which form protein complexes after their translation. The PER:CRY complexes downregulate *PER* and *CRY* transcription, which closes the loop. In another transcriptional-translational-feedback-loop, ARNTL:CLOCK activate the transcription of *NR1D1* and *NR1D2*. They are translated into gene regulatory proteins which inhibit *ARNTL* transcription, closing this loop. NR1D1 and NR1D2 proteins also inhibit *NFIL3* transcription. NFIL3 is part of further transcriptional-translational-feedback-loops, activating the transcription of *RORA, RORB* and *RORC*. The ROR proteins activate *NFIL3* and *ARNTL* transcription, resulting in positive and negative regulatory feedback.

Further regulators within the transcriptional-translational-feedback-loops of the circadian clock are *DBP, CIART, STAT3* and *BHLHE41*. The protein of *DBP* has similar binding sites to NFIL3 and regulates the transcription of *RORs, NR1Ds* and *PERs*. The CIART protein is another ARNTL:CLOCK repressor and the STAT3 protein another regulator of *DBP* and *NFIL3*. The protein BHLHE41, also called DEC2, reduces the gene expression levels of *PERs, CRYs, DBP* and *NR1D2*^4^.

The transcriptional-translational-feedback-loops cause oscillations of the clock gene transcript levels and their regulatory targets. This can be observed in gene expression time series data. Circadian oscillations in gene expression have a period of approximately 24 h and stable amplitudes. There are relatively fixed phase differences between the oscillations of different circadian expressed genes.

When healthy mammalian cells turn into cancer cells, the gene regulatory network of the circadian clock may be altered, resulting in modified or even abolished oscillations of the circadian expressed genes. The alterations of the transcriptional-translational-feedback-loops in many cancerous tissues are cancer specific^5^. In non-small-cell lung cancer, *PER2*^6^ and *RORA*^7^ are downregulated. A single nucleotide polymorphism in *PER3* is a risk factor for the development of the disease^8^ and low levels of *PER1*, *PER2* and *PER3* are associated with a poor prognosis^9^. For squamous-cell lung cancer, high levels of *CRY2, ARNTL* and *RORA* transcription and low levels of *NPAS2* transcription indicate a favourable prognosis^10^. Low levels of *NPAS2* also improve the survival prognosis in lung adenocarcinoma^5^.

The investigation of the gene regulatory network of the circadian clock of cancerous human tissue is challenging. All cancer samples are acquired during surgeries that are not part of an experimentally planned gene expression time series. Also, the samples are often unannotated with collection times when they are uploaded into public databases, e.g., ’The Cancer Genome Atlas’ (TCGA)^11^ (Table 1).

There are multiple methods to overcome this by estimating temporal labels of non-circadian gene expression samples. Zeitzeiger^12^, BodyTime^13^, TimeSignature^14^ and TimeTeller^15^ are trained with gene expression time series data of the same tissue, or the same species, to get small circadian gene sets (12–3 genes, see Table 2) that give good predictions of circadian time. The dataset-specific training data has to be collected in advance and is not available for human tissues types for which samples have to be collected invasively.

In contrast, unsupervised circadian time assignment methods are applied to large amounts of temporally unannotated gene expression data or other -omics data^16^ without prior training on a gene expression time series. The set of genes that they unsupervisedly learn to represent circadian time in a dataset is not limited to circadian genes. A well-known example is the circular autoencoder CYCLOPS^17^ that was used for a rhythmicity analysis of non-cancerous human lung biopsy data as well as for the investigation of differential clock gene expression of healthy and cancerous liver tissue. Further unsupervised circadian time assignment studies linked circadian clock strength with courses of breast cancer^18^ or used GTEx data to provide a list of 12 genes (Table 2) with well-conserved phase differences between clock gene expressions in 46 healthy human tissues^19^.

Alternatively, it is also possible to analyze phase relationships between circadian genes without estimating sampling times. Shilts et al.^20^ used the Spearman rank correlations between 12 well-known core clock genes (Table 2) to compute clock gene correlation matrices of non-circadian public gene expression datasets of healthy and cancerous tissues and found disturbed circadian rhythms in multiple cancer types. The 12 genes were complemented with *NFIL3, BHLHE41, RORC, CIART* and *HLF* to investigate the effect of non-clock pathways, including cell cycle, on the circadian clock in human epidermis, melanoma, pancreatic cancer and other cancers^21–23^. Likewise, Wu et al.^24^ used a gene list of 10 core clock genes (Table 2) on public human gene expression data and found lower coefficients of variation when they compared cancer samples and normal samples in lung cancer (TCGA-LUAD, GEO-LUAD), kidney cancer (TCGA-KIRC, GEO-KIRC), thyroid cancer (TCGA-THCA), liver cancer (TCGA-LIHC), breast cancer (TCGA-BRCA), and prostate cancer (TCGA-PRAD). They claim that clock gene oscillations are not very robust and have low relative amplitudes in these cancers.

In our study, we wanted to compare the strength of the circadian clock in healthy and cancerous lung tissue. For a strong circadian clock, we expected stable and gene-specific phase differences between the oscillating gene expression levels of circadian-expressed genes. Based on their correlations to the circadian gene *PER3*, we obtained lists of circadian-expressed genes in high-throughput gene expression time series data of lung tissue from differently aged mice^25,26^ and baboon^27^. Then we analysed the phase relationships, the rhythmicities, and the amplitudes of the gene expression levels of the genes in our circadian gene lists. We used these properties to compare the mammalian circadian gene regulatory networks of mouse lung and baboon lung to TCGA-LUAD data from healthy and cancerous human lung tissue.

We tested the quality of the representation of the mammalian circadian clock gene regulatory network in our gene lists by PCA-based reconstructions of the circadian time of the temporally annotated gene expression samples of mouse and baboon. Then we used a shared mammalian circadian gene list of 13 genes to assign potential circadian times to the temporally unannotated human lung tissue samples from the TCGA-LUAD study. When we compared the reconstructed oscillations of the clock gene expression in healthy and cancerous lung tissue, we observed weak oscillations of the clock gene expressions in lung cancer. The phase differences between the clock gene oscillations appeared to be altered for many clock genes in lung cancer.

## Results

### Correlations between circadian expressed genes contain information about the phase differences between their oscillating transcript levels

**Figure 1:**
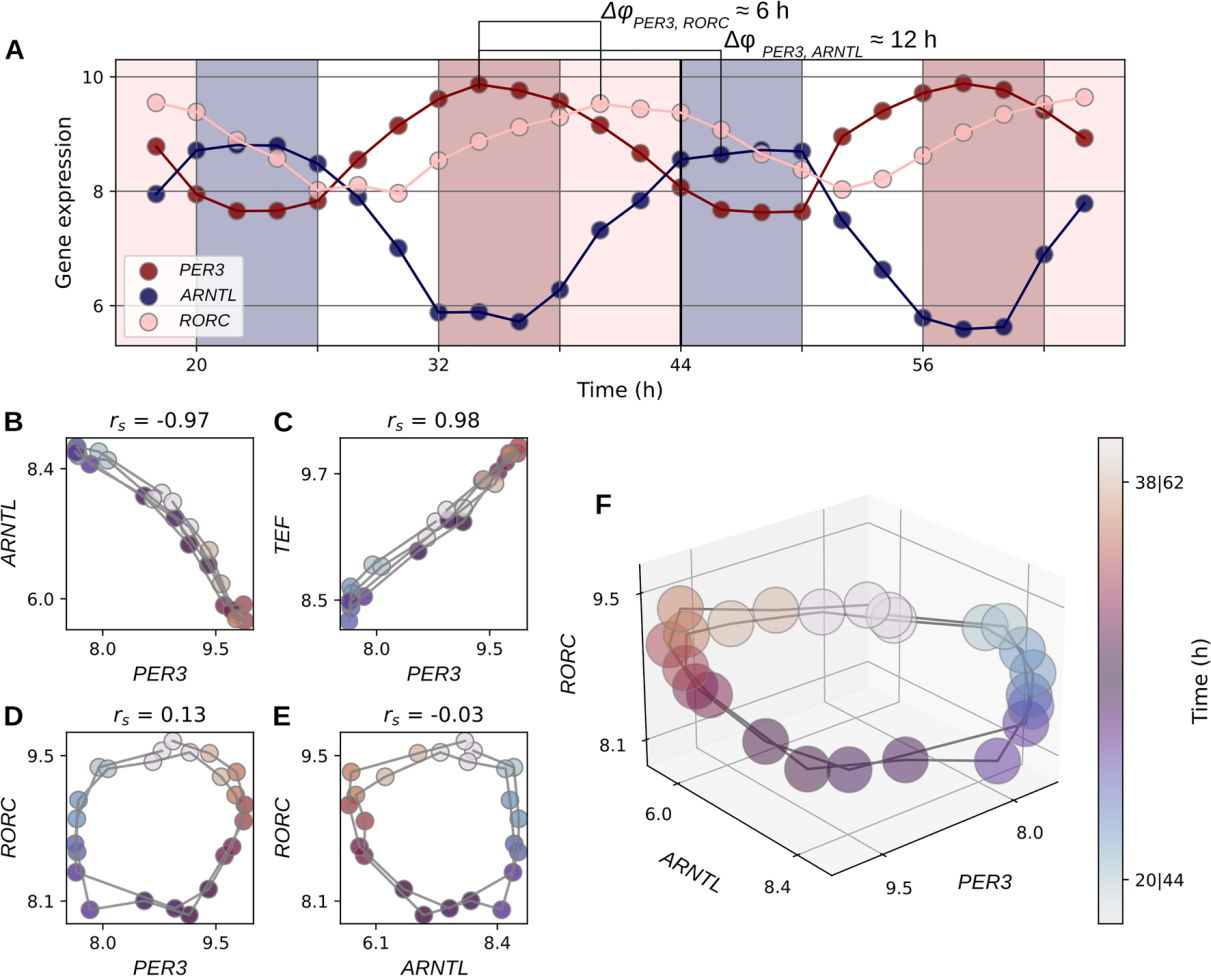
Circadian gene expression levels form circles in gene expression space (**A**) Circadian oscillations of *PER3* (red), *ARNTL* (blue) and *RORC* (light pink). There is a 6-h phase difference between *PER3* and *RORC* and a 12-h phase difference between *PER3* and *ARNTL*. Blue bars mark subjective night, white bars mark subjective dawn, red bars mark subjective day and light pink bars mark subjective dusk. (**B**) Negative correlation between *PER3* and *ARNTL*. (**C**) Positive correlation between *PER3* and *TEF*. (**D**–**F**) Circular relationship between *PER3*, *RORC* and *ARNTL*. The colors in B-F indicate sample collection times. All plots show gene expression data of mouse lung tissue from Zhang et al. as log2(counts).

In this study we compare four gene expression datasets (Table 1): two lung datasets from murine multi-organ studies^25,26^, one lung dataset from a multi-organ study of baboon^27^ and data of healthy and cancerous lung rissue from the TCGA-LUAD study. The datasets of Zhang, Wolff and Mure are gene expression time series of 12 (Mure and Wolff) or 24 (Zhang) lung tissue samples. These samples were collected throughout the day and are annotated with their sampling times. In contrast, the TCGA-LUAD study did not have a circadian focus and the samples do not have any time labels. It includes 51 normal samples and 58 tumor samples, each normal sample is matched with at least one tumor sample of the same patient.

Figure 1A shows the circadian rhythms of *PER3, ARNTL* and *RORC* transcript levels over two days in mouse lung gene expression time series data from Zhang et al.^25^. The transcript levels of *PER3, ARNTL* and *RORC* oscillate with a 24-h period and their circadian transcript level oscillations have stable amplitudes. Within each 24-h period, there are fixed time differences, referred to as phase differences ($\Delta \varphi$), between the times of the transcript level peaks. The phase differences are gene pair specific. For example, there is an approximate 12-h phase difference between *PER3* and *ARNTL* and an approximate 6-h phase difference between *PER3* and *RORC* (Fig. 1A).

The phase differences between circadian expressed genes are linked tightly to the Spearman rank correlation between their transcript levels ($r_{S}$) and this $\Delta \varphi$-$r_{S}$-relationship generally applies to pairs of sine curves (Supplementary Fig. S1). Circadian expressed genes whose transcript levels oscillate with an approximate 12-h phase difference, e.g., *PER3* and *ARNTL*, have a negative linear relationship of their transcript levels (Fig. 1B) with a strong anti-correlation ($r_{S} \approx$ − 0.97). Likewise, circadian expressed genes whose transcript levels oscillate with an approximate zero-hour phase difference, e.g., *PER3* and *TEF*, have a positive linear relationship of their transcript levels (Fig. 1C) and a strong positive correlation ($r_{S} \approx 0.98$).

There are also several circadian expressed genes whose transcript levels oscillate with intermediate phase differences between zero hours and 12 h. This results in ellipsoid transcript level relationships with low correlation values. In the special case of an approximate 6-h phase difference between the transcript level oscillations, e.g., *PER3* and *RORC* or *ARNTL* and *RORC*, the pattern in their shared gene expression space is circular (Fig. 1D,E). Therefore, the correlations between the transcript levels of *PER3* and *RORC* ($r_{S} \approx 0.13$) or *ARNTL* and *RORC* ($r_{S} \approx$ − 0.03) are almost zero.

For an intact circadian clock, the oscillating transcript levels of all circadian expressed genes form a high dimensional cycle in gene expression space. Figure 1F shows the three-dimensional cycle in the shared gene expression space of *PER3, RORC* and *ARNTL*. The cycle is colored for the collection times of the samples. Like in a phase portrait^28^, each location on this cycle in circadian gene expression space encodes another circadian time.

### Dataset-specific lists of phase-sorted circadian genes based on their transcript-level correlations

**Figure 2:**
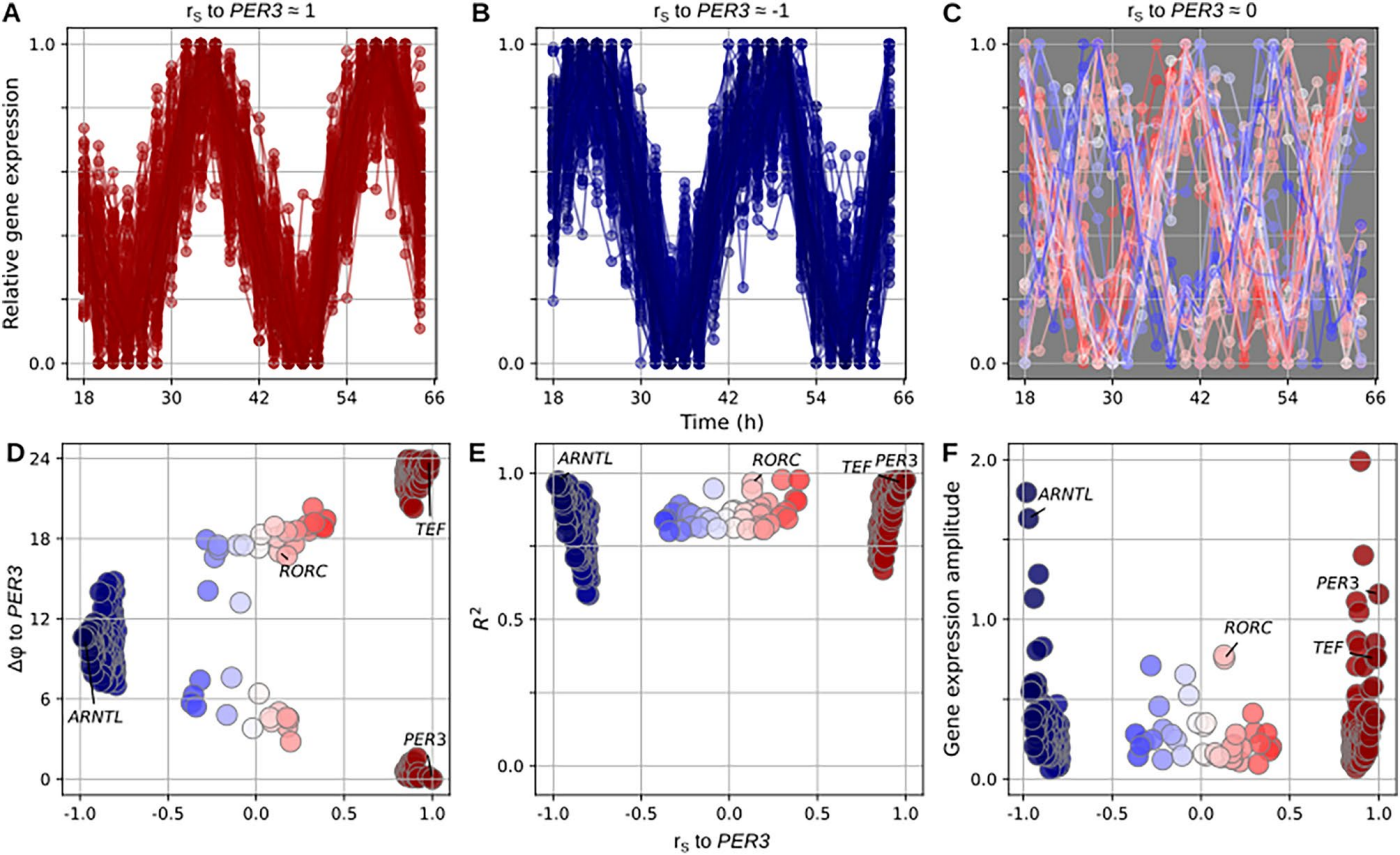
Phase-sorted circadian genes in mouse lung (**A**) Normalized circadian oscillations of 100 genes that oscillate in-phase to *PER3*. (**B**) Normalized circadian oscillations of 100 genes that oscillate out-of-phase to *PER3*. (**C**) Normalized circadian oscillations of genes that oscillate with a 6-h phase difference to *PER3*. (**D**) Relationship between Spearman rank correlations and phase differences between the oscillations of *PER3* and the genes in (**A**–**C**). (**E**,**F**) Relationship between the Spearman rank correlation, regression accuracies $R^{2}$ and amplitudes of the genes from (**A**–**C**). All plots show gene expression data of mouse lung tissue from Zhang et al.

The oscillating transcript levels of all circadian expressed genes within the same tissue form a high dimensional cycle in gene expression space (Fig. 1F shows a three-dimensional example from the Zhang dataset). Also gene expression samples that do not originate from gene expression time series data and are not annotated with collection times form this cycle in circadian gene expression space. The spatial position of the samples in the cycle gives information on their circadian time. We expect that the analysis of a high number of circadian expressed genes can give a good representation of the circadian cycle. For each mouse dataset and for the baboon dataset, we used the relationship of phase differences and transcript level correlations of circadian expressed genes to find species- and age-specific lists of circadian expressed genes that give a good representation of the circadian cycle.

We chose *PER3* as circadian starting gene because it has a reliable circadian expression^19^ and computed the Spearman rank correlations, $r_{S} \in [-1, 1]$, between the transcript level of *PER3* and the transcript levels of all other genes. Then we sorted all genes for $r_{S}$ and chose the top 100 and the bottom 100 genes for dataset specific circadian gene lists. This results in two groups of circadian expressed genes in each list, which differ by their phase difference ($\Delta \varphi$) to *PER3*. The transcript levels of the bottom 100 genes oscillate out-of-phase to *PER3* ($r_{S} < -0.7$ and $\Delta \varphi \approx 12$ h in Fig. 2B) and the transcript levels of the top 100 genes are in-phase to *PER3* ($r_{S} > 0.7$ and $\Delta \varphi \approx 0$h in Fig. 2A). Both gene groups contain known clock genes and genes who are known for being regulated by clock genes. The quality of the circadian rhythmicity of the transcript levels can be quantified by $R^{2}$ of a harmonic fit, which shows that the transcript level oscillations of many genes in both gene groups are very circadian ($R^{2} > 0.5$ for $r_{S} \approx \pm 1$ in Fig. 2E). There are some circadian genes in the lists whose transcript level oscillations have high amplitudes (Fig. 2F).

Unfortuntaly, the in-phase and out-of-phase gene groups are not sufficient for a complete representation of the different times of the circadian day^29^ in gene expression space. The transcript levels of the in-phase gene group peak during subjective day, while the out-of-phase genes have a transcript level trough. Thus, a temporally unlabelled gene expression sample with high transcript levels of the in-phase gene group and low levels of the out-of-phase gene group can be recognised as a sample from subjective day (red time bars in Fig. 1A). Similarly, the transcript levels of the in-phase gene group have a trough at subjective night, while the out-of-phase transcript levels peak, and a temporally unlabelled sample with this gene expression pattern can be assigned to subjective night (blue time bars in Fig. 1A). Finally, a sample with intermediate transcription levels of both gene groups can belong subjective dawn or subjective dusk. The gene expression pattern is not sufficient to distinguish between these two times of the day, which are 12 h apart from each other (white vs light pink time bars in Fig. 1A).

The time assignment ambiguity can be resolved when we include additional circadian genes, whose oscillating transcript level peak 6 h ($\Delta \varphi$ = 6h, light pink curve in Fig. 1A) after the peak in *PER3* transcript level. These genes have their peak in transcript levels while the in-phase gene groups’ ($\Delta \varphi$ = 0 h) transcript levels are falling, and they have a trough in transcript levels when the in-phase gene groups’ ($\Delta \varphi$ = 0h) transcript levels are rising. A temporally unlabelled gene expression sample in which the transcript levels of the in-phase and out-of-phase genes are close to their mesors will have either a peak or a trough in the transcript levels of the 6-h phase difference genes and can therefore be unambiguously assigned to either subjective dawn or subjective dusk.

As we have shown in Fig. 1, the addition of a 6-h-phase-difference gene group improves the representation of the circadian cycle and the circadian gene regulatory network in the dataset-specific circadian gene lists. Circadian genes whose transcript levels oscillate with a 6-h phase difference to each other have circular relationships in gene expression space, which result in almost zero correlations of the transcript levels (Fig. 1C,E). However, we cannot find genes with a six-hour phase difference to *PER3* solely on the basis of a zero correlation ($r_{S} \approx$ 0) between their transcript levels and the *PER3* transcript level, because also non-circadian genes have vanishing correlations. This is why we find the 6-h-phase-difference gene group by choosing genes with low correlations of their transcript levels to the transcript levels of *PER3* and *ARNTL* ($-\,0.4< r_{S} < 0.4$ in Fig. 2D) and good circadian rhythm ($R^{2} > 0.8$ in Fig. 2E) of these genes’ gene expression time series. The rhythmicity threshold excludes non-oscillatory expressed genes. The obtained $\Delta \varphi$ = 6 h gene group consists of genes which have their peak in transcript level either 6 h before or 6 h after the peak of the *PER3* transcript level (Fig. 2C,D). Figure 2D shows the generic relationships between rank correlation coefficients and phases of circadian genes. It confirms that we can infer phase relationships from the correlation patterns of those genes.

We get five lists with $\Delta \varphi$ = 0 h, $\Delta \varphi$ = 12 h, and $\Delta \varphi$ = 6 h gene groups for the five gene expression time series datasets of lung tissue of mouse and baboon (five supplementary Tables). Then we get two shorter circadian gene lists by choosing genes which are shared across these lists of lung genes. The short lists are then applied to study gene expression snapshots of human lung tissue.

### Phase differences between the transcript levels of Per3 and 12 other circadian genes are conserved in the lung tissues of mouse, baboon and human

**Figure 3:**
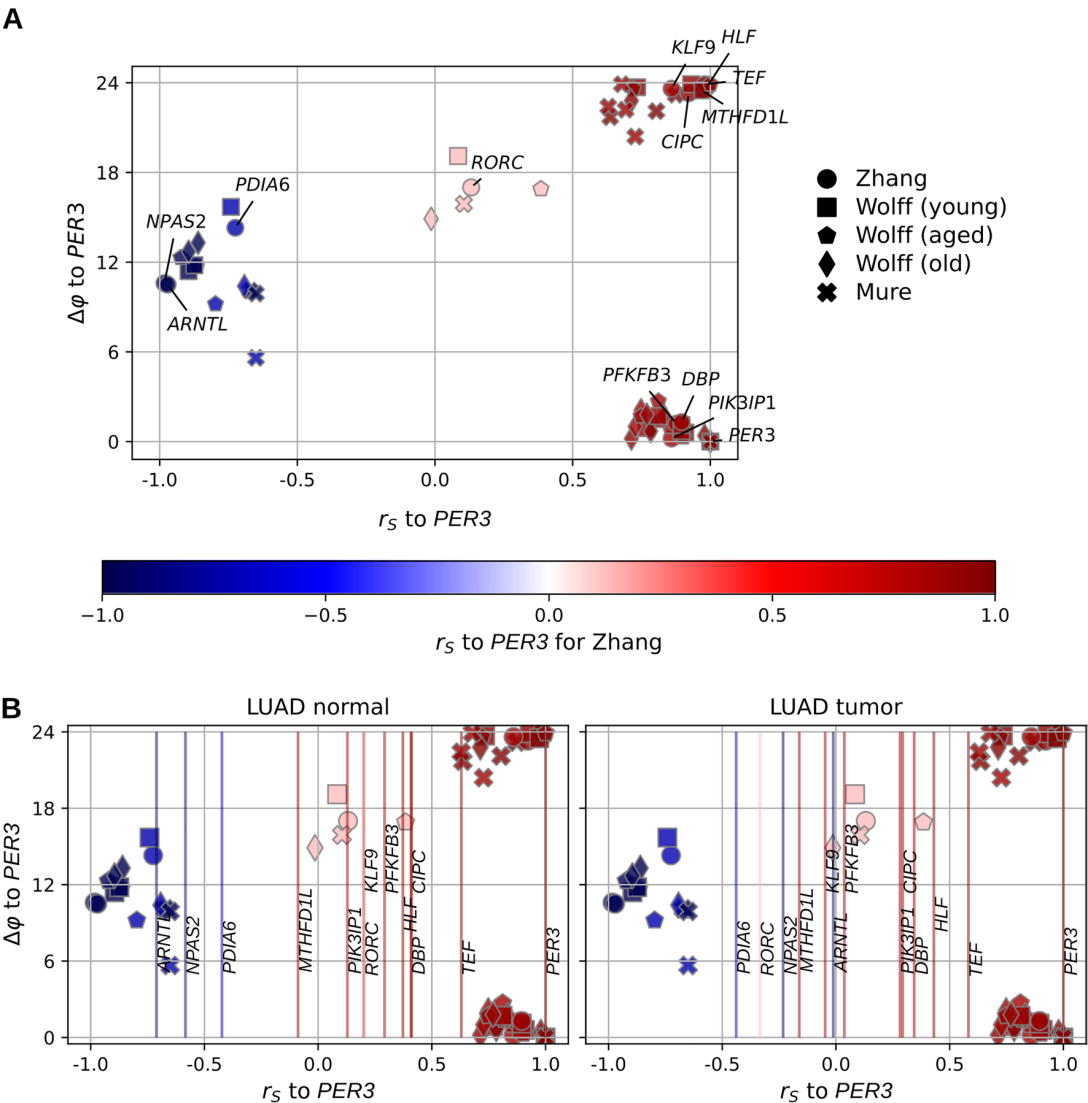
Comparison of conserved clock gene phase relationships in mouse and baboon lung to clock gene relationships in normal/cancerous human lung tissue (**A**) Relationship between the Spearman rank correlation and the phase difference between the oscillations of *PER3* and our 13 circadian mammalian lung genes for gene expression time series data of mouse lung (Zhang, Wolff) and baboon lung (Mure). Marker symbols indicate gene expression datasets. (**B**) Spearman rank correlations of *PER3* and our 13 circadian mammalian lung genes for mouse lung, baboon lung and healthy (left) and cancerous (right) human lung tissue from TCGA-LUAD. The human gene expression snapshots from TCGA-LUAD allow a calculation of Spearman rank correlations, but not of phase differences between circadian genes. Therefore the human Spearman rank correlations are plotted as lines that cross all possible phase differences. The data in (**A**) is repeatedly shown for comparison and the line colors correspond to the gene labels in (**A**).

**Figure 4:**
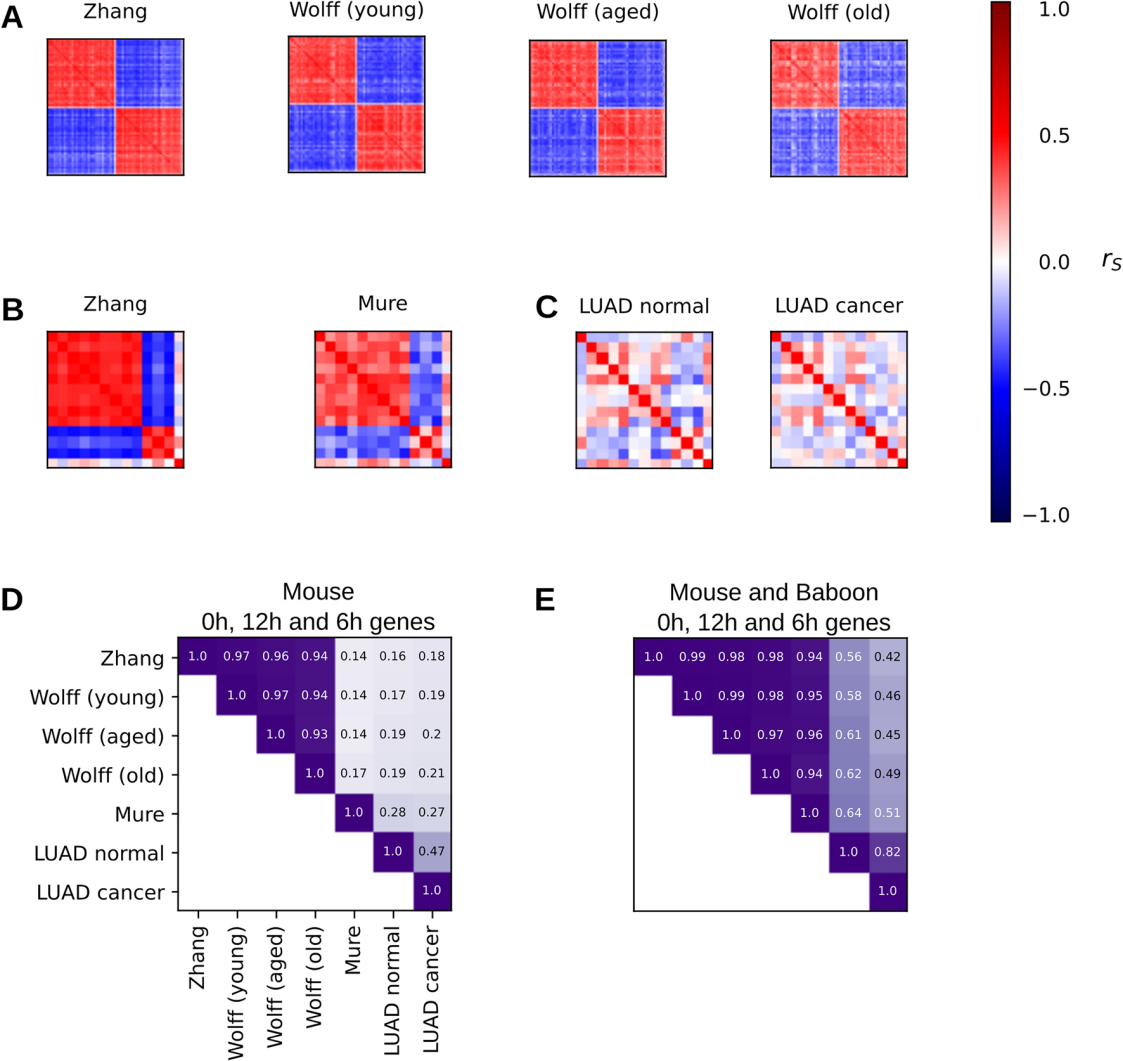
Circadian gene correlations in mouse and baboon lung and normal/cancerous human lung tissue (**A**) Spearman rank correlations between all 107 genes of the shared murine circadian gene list for mouse lung gene expression data from Zhang et al. and Wolff et al. The complete caption of the horizontal and vertical axes is given in Table 3. (**B**,**C**) Spearman rank correlations between all 13 genes of the shared mammalian circadian gene list for gene expression data of mouse lung (from Zhang et al.), baboon lung (from Mure et al.) and TCGA-LUAD. (**D**) Cosine similarity of the correlation matrices of all aforementioned datasets for the shared murine circadian gene list and (**E**) the shared mammalian circadian gene list.

By applying our gene selection exemplified in Fig. 2, we obtained four specific lists of circadian expressed genes in mouse lung. There is one list for the data from^25^ and three lists for the data from^26^, with mice of three different age groups. For a comparison of the circadian gene regulatory networks of all mouse datasets we combined all four gene lists into a shared murine circadian gene list of 107 genes (Table 3). It includes all genes, which are present in all four murine circadian gene lists. The resulting murine circadian gene list still consists of three gene groups, whose transcript levels oscillate with three different phase differences ($\Delta \varphi$ = 0 h, $\Delta \varphi$ = 12 h, or $\Delta \varphi$ = ± 6 h) to the oscillating transcript levels of *PER3*. In the 6-h phase difference gene group, only *RORC* and *CRY1* were shared across all our murine circadian gene lists. The 12-h phase difference gene group contains 52 genes, which have their peak in gene expression 8.4–15.6 h before the peak in *PER3* gene expression. In the zero-hour phase difference gene group are 53 genes, which have their peak in gene expression 3.3 h before to 2.5 h after the peak in Per3 gene expression (Fig. 4Supplementary Fig. S2).

We wanted to get a circadian gene set, which can give information on the circadian clock also in non-murine mammalian lung tissue. Therefore, we constructed a shared mammalian circadian gene list of lung tissue of mice and baboon by choosing all homologous genes, which intersect between the shared murine circadian gene list and our circadian gene list of the baboon dataset. The resulting list consists of 13 genes (Table 3). There are eight genes (*MTHFD1L, PIK3IP1, HLF, CIPC, TEF, KLF9, PFKFB3, DBP*), whose transcript level peak 4.1 h before to 2.9 h after the peak in *PER3* transcript level. Three genes (*NPAS2, PDIA6* and *ARNTL*) have their peak in transcript level 7.5–18.7 h before the peak in *PER3* transcript level. *RORC* is the only remaining gene of the 6-h phase difference gene group (Fig. 3A).

### Correlations between circadian genes in TCGA-LUAD snapshot data shows differences between healthy and cancerous TCGA-LUAD samples

We compare now the mammalian circadian gene regulatory networks in mouse lung and baboon lung to the circadian gene regulatory networks of healthy and cancerous human lung tissue from TCGA. Unlike the gene expression time series data from mouse and baboon, the TCGA samples are temporally unlabelled gene expression snapshots. Without temporal annotation of the TCGA data it is impossible to compute any phase differences, $\Delta \varphi$, of circadian transcript oscillations. However, it is still possible to compute Spearman rank correlations $r_{S}$ between the transcript levels of the gene expression snapshots.

We computed the Spearman rank correlations, $r_{S}$, between the transcript level of *PER3* and the transcript levels of the genes of the curated circadian gene list for healthy and cancerous human lung tissue samples. Then we marked these $r_{S}$ as axis intersections in the $r_{S}$-$\Delta \varphi$-plot of the mouse data and the baboon data (Fig. 3B). When the $r_{S}$-line of a circadian gene is located closely to the $r_{S}$-$\Delta \varphi$-cluster of mouse and baboon of the same gene, it is likely that the $\Delta \varphi$ of this gene is similar to the $\Delta \varphi$ in mouse and baboon. This is the case for many $r_{S}$-lines of circadian genes in the healthy adjacent lung tissue samples from the TCGA-LUAD study (Fig. 3B).

In the cancerous lung tissue of the TCGA-LUAD samples, the circadian clock is altered. There is a strong alteration in the circadian behaviour of *ARNTL* and *NPAS2*, which are both shifted closer towards $r_{S} = 0$ (Fig. 3B). Additionally, *RORC* is shifted from $r_{S} \approx 0.2$ to $r_{S} \approx -0.4$. In contrast, the correlations between *PER3, TEF, HLF and DBP* are not altered in lung cancer (see also Supplementary Fig. S3C). If *ARNTL* or *NPAS2* are chosen as reference gene instead of *PER3*, the $r_{S}$ of all genes are densely located around $r_{S} \approx 0$ in the cancerous TCGA-LUAD data (Supplementary Fig. S3A+B).

### Weak correlation pattern of circadian expressed genes in human lung cancer

Circadian correlation matrices (CCM)^12^ provide an overview of the pairwise correlations between the gene expressions of all genes of a given circadian gene list. As the correlations between the expressions of circadian genes are closely linked to the phase differences between their oscillations, they also provide insight into all the phase differences between these genes. The calculation of circadian correlation matrices is also possible for gene expression snapshot data, allowing us to compare the circadian expressed genes in mouse and baboon lung tissue to their homologs in human lung tissue from TCGA-LUAD. We computed circadian correlation matrices for the shared murine circadian gene list (Fig. 4A) and the shared mammalian gene list (Fig. 4B,C). We also used cosine similarities (Equation 4) to quantify the strength of the differences between the circadian correlation matrices (Fig. 4D) of the different datasets for the same gene list.

The circadian gene expression matrices of the shared murine circadian gene list show a clear separation of the two large gene groups with $\Delta \varphi$ = 0 h and $\Delta \varphi$ = 12 h (Fig. 4A). There is a high conservation of this correlation pattern across all murine datasets. Accordingly, the cosine similarities of the CCMs of the shared murine circadian gene list are very high for all mouse datasets, while the cosine similarities between the CCMs of mouse and baboon and of mouse and human are much lower (Fig. 4D). This might indicate the presence of mouse specific circadian genes or mouse specific phase relationships with low variability between mouse datasets.

For the shared mammalian circadian gene list, a pattern of distinct $\Delta \varphi$ = 0 h, $\Delta \varphi$ = 12 h, and $\Delta \varphi$ = ± 6 h genes is seen in the circadian correlation matrices (Fig. 4B) of mouse and baboon, indicating a high similarity between their circadian rhythms. This pattern is similar, but altered in the circadian correlation matrix of the healthy human lung data (Fig. 4C). More specifically, the oscillatory gene expressions of *MTHFD1L, KLF9* and *PFKFB3* from the $\Delta \varphi$ = 0 h gene group have potential phase shifts in healthy human lung, but the oscillations of the transcripts of *PIK3IP1, HLF, CIPC, TEF, PER3, DBP*, the $\Delta \varphi$ = 12 h and the $\Delta \varphi$ = ± 6 h genes might have similar phases as in mouse and baboon. Accordingly, the cosine similarities of the circadian correlation matrices of the shared mammalian circadian gene list are higher when healthy human lung tissue is compared to mouse and baboon than when cancerous human lung tissue is compared to mouse and baboon (Fig. 4E). However, the similarity of healthy human lung tissue to cancerous human lung tissue is higher than the similarities of healthy human lung tissue to mouse and baboon lung tissue. This can result from human-specific phase deviations of the circadian genes. Additionally, the healthy human lung tissue was collected in vicinity of the tumor tissue, which could also have increased its similarity to the tumor tissue.

Overall, the phase differences between our 13 circadian genes from the gene regulatory network of the circadian clock in mammalian lung tissue have a high conservation in mouse and baboon. Many of these phase differences are also conserved in the healthy human lung tissue samples of the TCGA-LUAD study, but there is a potentially altered circadian clock in the cancerous human lung tissue samples of the TCGA-LUAD study.

### PCA based reconstruction of circadian gene expression rhythms in the TCGA-LUAD dataset shows altered circadian rhythms in human lung cancer

**Figure 5:**
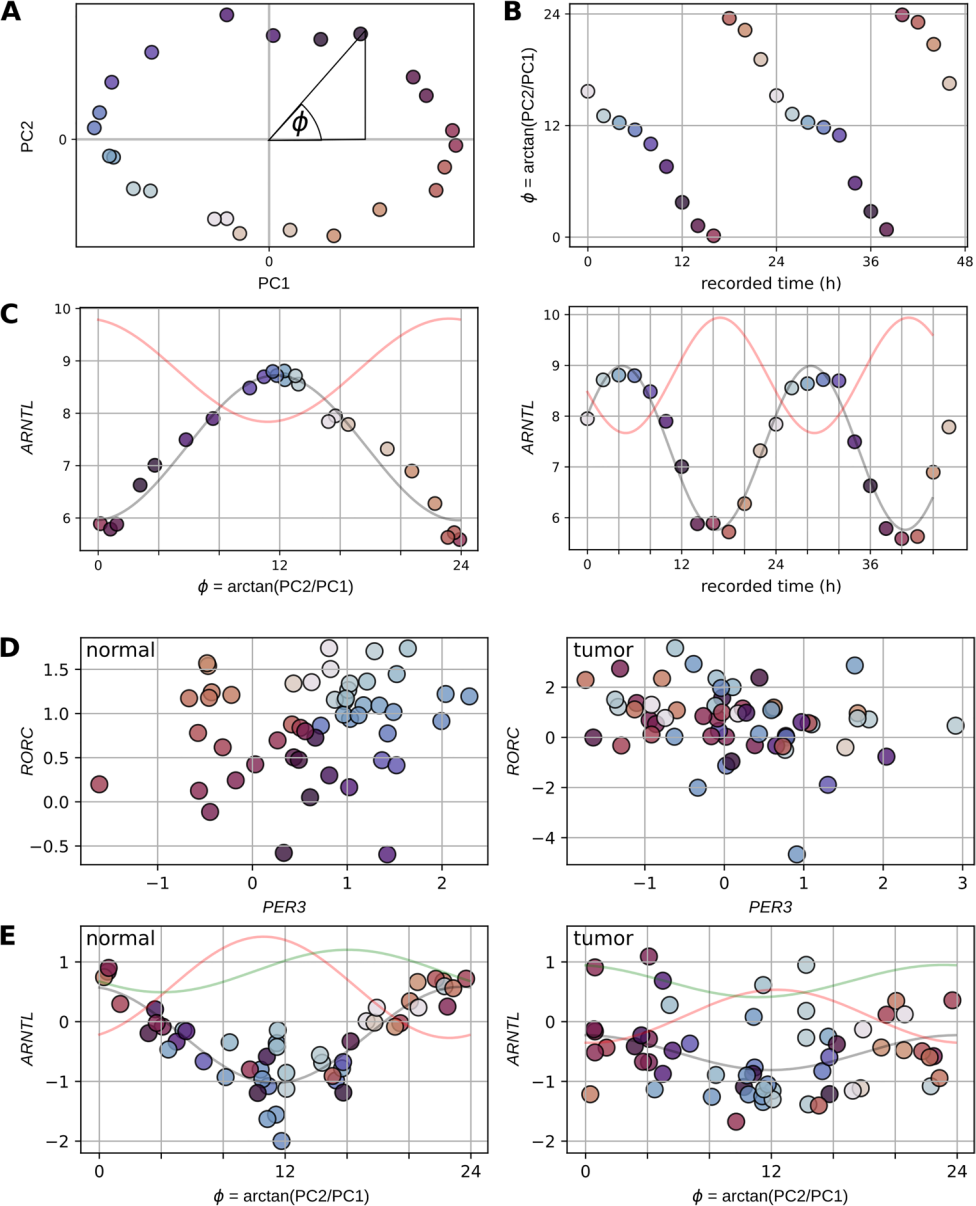
PCA-based reconstruction of circadian gene expression in mouse lung and normal/cancerous human lung tissue (**A**) Circular relationship between PC1 and PC2 after a PCA of the gene expressions depicted in Fig. 2. (**B**) Linear relationship between recorded time and $\phi$. (**C**) Reconstructed and recorded oscillations of *ARNTL* (black line and circles) and *PER3* (red line). (**D**) Relationship between *PER3* and *RORC* in paired normal/tumor samples. (**E**) Reconstructed oscillations of *ARNTL* (black line and circles), *PER3* (red line) and *RORC* (green line) in paired normal/tumor samples using the shared mammalian gene list. (**A**,**B**,**C**) show mouse lung data from Zhang et al. (**D**,**E**) show TCGA-LUAD data.

We test the representation of the mammalian circadian clock gene regulatory network in our gene lists using Principal Component Analysis (PCA) based reconstructions of the circadian times of temporally annotated mouse and baboon gene expression samples. The principal components (PCs) of a multidimensional gene expression dataset are the combinations of gene expressions with the greatest variance.

A PCA of the transcript levels of our selected dataset-specific circadian genes for the mouse dataset of^25^ results in a circular relationship of the first and second principal components (Fig. 5A). The spatial position of the gene expression samples on the circle is related to their collection time (colour coded in Fig. 5A+B). More specifically, there is a linear relationship between the collection time and the angle on the circle $\phi = arctan\left( \dfrac{PC2}{PC1}\right)$ with $\phi \in [0, 24]$h. The reconstructed circadian rhythms of the transcript levels of *ARNTL* (black line in Fig. 5B) and *PER3* (red line in Fig. 5B) match the recorded gene expression time series. A successful reconstruction of the circadian gene expression time series is also indicated by the fact that the reconstructed and recorded transcript levels of *PER3* and *ARNTL* have the same phase difference $\Delta \varphi$.

We see the circular relationship of the first and the second principal component for the application of each of our circadian gene lists on the mouse datasets and the baboon dataset (Supplementary Fig. S4). The mouse data from^26^ includes groups of mice of three different ages. We combined them into one group to get a more heterogeneous dataset that might be more similar to human gene expression data collected in the clinic. Then we used the first and second principal component of the gene expression levels of the genes from our shared mammalian circadian gene list from this dataset to reconstruct its gene expression time series. There is a good time series reconstruction for this test case (Supplementary Fig. S5), which supports a good representation of the circadian gene regulatory network of the mammalian lung in this genelist.

In order to quantify the strength of the circadian clock in lung cancer, we used the shared mammalian circadian gene list (Table 3) to assign potential circadian times to 51 temporally unannotated healthy human lung tissue samples from the TCGA-LUAD study. There are no time labels available to give a direct indication of the quality of the reconstruction. However, there is a circular relationship between the transcript levels of *PER3* and *RORC* for an intact mammalian clock. We used $\phi = arctan\left( \dfrac{RORC}{PER3}\right)$ as proxy for the true circadian time (left plot in Fig. 5C). The phase differences between the reconstructed gene expression time series of *ARNTL* ($R^{2} \approx$ 0.76, blue line in Fig. 5D), *PER3* ($R^{2} \approx$ 0.61, red line in Fig. 5D) and *RORC* ($R^{2} \approx$ 0.19, green circle in Fig. 5C) are consistent with the expected phase differences between *ARNTL, PER3* and *RORC* for a healthy mammalian circadian clock (Supplementary Fig. S6).

Next, we used the reconstructed circadian times of the normal samples from the TCGA data to temporally sort their 58 paired adjacent tumour samples. We observe that the circadian rhythms of the transcript levels of *PER3* ($R^{2} \approx$ 0.11), *ARNTL* ($R^{2} \approx$ 0.11) and *RORC* ($R^{2} \approx$ 0.02) are weakened in lung cancer (Supplementary Fig. S6). Additionally, the phase difference between the circadian rhythms of the transcript levels of *PER3* and *RORC* is increased by 8 h in the cancerous lung tissue. This indicates an alteration of the gene regulatory network of the circadian clock in lung cancer.

## Discussion

In this study, we compared the gene regulatory networks of the circadian clock in the lung tissue from mice^25,26^ and baboons^27^ with the gene regulatory networks of the circadian clock in about 50 paired samples of healthy and cancerous human lung tissue from TCGA-LUAD.

We derived dataset specific lists of circadian expressed genes in lung tissue of differently aged mice^25,26^ and baboons^27^. These circadian gene lists should include genes with circadian rhythms that are robust enough to be also present in cancerous lung tissue. We did not want to use a fixed, literature based core clock gene list, because such a gene list could lack lung tissue specific circadian genes. However, many genes in our shared mammalian circadian gene list are also well-known core clock genes.

To create the gene lists, we used transcript level correlations to select circadian genes, that have a daily peak in transcript level with 0-hour, 12-h or 6-h time difference to the daily peak in *PER3* transcript level. The 6-h gene group ensures that for each quarter of the circadian day there are some transcripts in the list that are peaking or troughing. In the data considered in this study, the 6-h genes were rare. Mechanistically, the time differences between the gene expressions peaks of circadian genes are determined by gene-specific presences of E-boxes, D-boxes and Rev-Response-Elements in these genes’ promoters^1,30^. *CRY1* and *RORC* have a 6-h phase difference to *PER3* and *ARNTL* in some or all of the examined datasets and they both contain an E-box and a Rev-Response-Element in their promoter. The strength of the gene regulation at the E-boxes determines whether these genes have a 6-h time difference in gene expression to *PER3*. This could be tissue and species specific, e.g. *CRY1* is part of the 6-h gene group in all examined mouse datasets, but not in the baboon dataset. Circadian genes that are very specific to human lung and thus not evolutionary shared with mouse and baboon, might be missing in our list.

There were 13 circadian genes that were present in all our dataset specific circadian gene lists. Although we selected the genes exclusively by their phase differences to *PER3*, there were strong conservations of the phase differences between all 13 circadian genes from the mammalian circadian gene regulatory network in mouse and baboon lung tissue.

If pre-mRNA expressions are also available for a dataset, the 13-gene list can be extended to include the pre-mRNA levels of the 13 genes. We expect that there will often be comparable but phase-shifted rhythms of mRNA and pre-mRNA levels of the same gene^31,32^. Thus, by adding the pre-mRNA levels, our list can cover more phase differences. This is can also be true for other -omics data^16^, covering further regulatory layers of the complex network of the mammalian circadian clock.

We expected that the molecular framework of the mammalian circadian clock is largely conserved across human, mouse and baboon lung tissue. Mure et al.^27^ already showed a conservation of the relative phases between many clock gene transcripts in gene expression time series data of lung tissue of mouse and baboon. Gene expression time series data from healthy human lung tissue is not available. However, clock gene phase differences in gene expression data from human skin^33^, skeletal muscle^32^ and immune cells^34^ largely match the phase differences between their expressed clock gene homologs in mouse and baboon and circadian transcript phases are largely conserved across different human tissues^19^.

Many phase differences that were conserved across mouse and baboon lung tissue were also conserved in the healthy human lung tissue samples of the TCGA-LUAD study. This supports an intact circadian clock in most of these samples. The high cosine similarities between the circadian correlation matrices of our 13-gene-list in healthy lung tissue of mouse, baboon and human support circadian clocks of comparable strengths in these datasets. In contrast, we found a reduced number of conserved phase differences and an altered circadian clock in the cancerous human lung tissue samples of the TCGA-LUAD study.

We confirmed the quality of the representation of the circadian gene regulatory network in mouse lung in our murine circadian gene list by using it for successful PCA-based reconstructions of the circadian time of the mouse gene expression samples. Then we used the shared mammalian circadian gene list to assign potential circadian times to the human lung tissue samples from the TCGA-LUAD study. We observed robust circadian oscillations of the transcript levels of the circadian genes in the reconstructed gene expression time series of healthy human lung tissue. In the cancerous human lung tissue, we observed weak circadian oscillations with low amplitudes and alterated phase differences between some of the transcript levels of the circadian genes. E.g., the circadian rhythms of *ARNTL, RORC* and *NPAS2* are potentially weak and the circadian rhythm of *RORC* might also be phase-shifted by several hours in lung cancer.

Overall, our work shows that it is worth to investigate the circadian gene regulatory network in archived and public cancer gene expression snapshot data from surgeries to gain insight into the circadian biology of cancer. We see that the analysis of circadian rhythms in gene expression data is facilitated, if a dataset covers a full circadian cycle. A fully covered circadian cycle also compensates for missing temporal labels, but clinical samples are collected during surgical working hours that are not evenly spread across the day. Fortunately, the natural availability of many different chronotypes and the random inclusion of shift workers^17^ in large patient derived cancer gene expression datasets rescue the representation of the circadian day. The increasing amount of publicly available human cancer gene expression samples will therefore facilitate future cancer-specific circadian gene expression studies. An interesting option to further test the robustness of correlation-based time allocation with incomplete temporal coverage of the circadian day in different human tissues is to also analyse circadian gene expression data from circadian entrainment experiments in cell cultures.

A way to integrate the results of this study into everyday clinical practice is to include the acquired 13-gene list of the mammalian circadian gene regulatory network into RNA sequencing panels of different cancer types to compare their circadian gene regulatory network with the circadian gene regulatory network in lung cancer. Knowledge of lung cancer specific alterations of the circadian clock may also help to improve circadian treatment schedules, which have already been shown to reduce side effects in lung cancer^35^.

## Methods

### Data availability

The datasets analysed during the current study are available in the repositories that are listed in Table 1.

**Table 1:** Analysed gene expression datasets.

Study	Species	Number of samples	Data access (accession no.)
Zhang et al.^25^	Mouse	24, Collected every 2 h	ncbi.nlm.nih.gov/geo (GSE54652)
			Apr 19 2022
Wolff et al.^26^	Mouse	12 Per age (young, aged, old), Collected every 4 h	ncbi.nlm.nih.gov/geo (GSE201207)
			Nov 10 2022
Mure et al.^27^	Baboon	12, Collected every 2 h	ncbi.nlm.nih.gov/geo (GSE98965)
			Apr 22 2022
TCGA-LUAD	Human	51 Normal, 58 tumor samples, Unrecorded collection times	portal.gdc.cancer.gov
			Mar 25 2022

We downloaded the gene expression counts of the four datasets from the sources listed in Table 1 and applied a log2 transformation to them if they were not already log2 transformed.

### Circadian gene lists

**Table 2:** Previously published mammalian circadian gene lists.

Reference	Circadian genes
Hughey et al.^12^	***DBP, PER3***, NR1D2, **TEF**, PER2, PER1, **NPAS2, ARNTL**, NR1D1, TSC22D3, LONRF3, *FMO2, CRY1*
Wittenbrink et al.^13^	*NR1D2*, ***PER3***, *NR1D1, LGALS3, PER2, ELMO2, FKBP4, HSPH1, CRY1, CRISPLD2,* ***KLF9***, PER1
Talamanca et al.^19^	***ARNTL***, CIART, CRY1, CRY2, **DBP, NPAS2**, NR1D1, NR1D2, PER1, PER2, **PER3** ***TEF***
Shilts et al.^20^	***ARNTL, NPAS2***, CLOCK, CRY1, CRY2, NR1D1, NR1D2, PER1, PER2, **PER3, DBP** ***TEF***
Wu et al.^24^	***ARNTL***, CLOCK, **NPAS2**, CRY1, NR1D1, CIART, **DBP**, PER1, CRY2, PER2

Genes that are also part of our mammalian circadian gene list are highlighted in bold.

**Table 3:** Our phase-sorted circadian expressed genes in murine and mammalian lung tissue.

Our Genelists	0 h	12 h	+-6 h
Mouse	*ALAS1, ARHGEF26, BHLHE40, BHLHE41*	*ACACA, ADAM17, ADM, ADRA1A*	*CRY1*
	*CCBE1, CEP85*, **CIPC**, *CLDN12, COQ10B*	*AHCYL2, AKR1E1, ALDH3A1*,*** ARNTL***	***RORC***
	*CPEB1, CYP3A13*, **DBP**, *FAM76A, FBXO3*	*AVPR1A, BIK, CALR, CCNJL, CDKN1A*	
	*GLUL, GPR155, GRAMD2, HDAC11*	*CXCL5, DAPK1, DPY19L3, DTX4, ELN*	
	*HERPUD1, HIF3A*, ***HLF***, ***KLF9***, *LPIN2*	*FAM124B, FAM210B, FOXS1, GDPD2*	
	*MACO1*, ***MTHFD1L***, *NANOS1, NR1D2*	*GLIPR2, GLRX, GPRIN3, HEATR1*	
	*OTUD1, PER1, PER2*, ***PER3, PFKFB3***	*IKZF4, INSYN1, LEO1, LMTK2, LPCAT3*	
	*PI4K2B*, ***PIK3IP1***, *POR, RAC3, RERE*	*MED24, NECTIN1, NFIL3*, ***NPAS2***	
	*REV1, RNASEH2B, SEPSECS, SIK1*	*P2RX5*, ***PDIA6***, *PFKP, PNRC1, RAB27A*	
	*SLC25A33, SLC46A3, SOCS2, SORD*	*RASL11A, RBM45, SCD1, SDF2L1*	
	*TRIM24, STK35*, ***TEF***, *TEX11, TSC22D3*	*SLC43A2, SNX30, SPAAR, SPON2*	
	*TSPAN4, USP2, WEE1*	*TMEM140, TMEM171, TMEM45A*	
		*TOR4A*	
Mammal	***MTHFD1L, PIK3IP1, HLF, CIPC, TEF*** ***KLF9, PFKFB3, PER3, DBP***	***NPAS2, PDIA6, ARNTL***	***RORC***

Genes that are also part of our mammalian circadian gene list are highlighted in bold. The gene lists are used as axes in Fig. 4A–C.

### Correlation analysis

We select genes that oscillate in-phase, anti-phasic or with 6-h phase difference to *PER3* for our circadian gene lists. There is a clear relationship between the phase differences $\Delta \varphi$ and the Spearman rank correlations $r_{S}$ of circadian genes, which we used for this selection. We used SciPy^36^ to compute all $r_{S} \in [-1, 1]$ as

1 $$\begin{aligned} r_{s} = \frac{cov[rank(x), rank(y)]}{\sqrt{cov[rank(x), rank(x)]cov[rank(y), rank(y)]}} \end{aligned}$$

using the sample covariance, *cov*(*x*, *y*), with the means $\bar{x}$ and $\bar{y}$:

2 $$\begin{aligned} cov(x, y) = \frac{1}{n - 1}\sum _{i=1}^{n} [x_{i} - \bar{x}][y_{i} - \bar{y}] \end{aligned}$$

### Rhythmicity analysis

We fit $X_{i}(t) = a*sin(\frac{2\pi }{24h}t) + b*cos(\frac{2\pi }{24h}t) + d*sin(\frac{2\pi }{12h}t) + e*cos(\frac{2\pi }{12h}t) + f$ to all gene expression time series $x_{i}(t)$ for computing phase differences between transcript level oscillations as peak to peak differences. Additionally, we compute the coefficients of determination $R^{2}$ of these fits as quality measure of the circadian oscillations.

3 $$\begin{aligned} R^{2}(x_{i}(t), X_{i}(t)) = 1 - \frac{\sum (x_{i}(t) - X_{i}(t))^{2}}{\sum (x_{i}(t) - \bar{x}_{i}(t))^{2}} \end{aligned}$$

Genes whose transcript levels have $-0.4< r_{S} < 0.4$ to *PER3* and *ARNTL*, and which have $R^{2} > 0.8$ are selected for the 6-h phase difference gene group.

### Cosine similarity for comparison of circadian correlation matrices

We use cosine similarities $S_{c}$ on the flattened circadian correlation matrices to compare their similarity across datasets.

4 $$\begin{aligned} S_{c}({\textbf {x}}, {\textbf {y}}) = \frac{\sum _{i=1}^{n}x_{i}*y_{i}}{\sqrt{\sum _{i=1}^{n}(x_{i}^{2})}*\sqrt{\sum _{i=1}^{n}(y_{i}^{2})}} \end{aligned}$$

All shared murine circadian genes that were not available in the baboon dataset were ignored for the computation of the cosine similarities.

### PCA and phase reconstruction

We use the PCA implemetation of scikit-learn^37^ to obtain the first two principal components of the transcript levels of our circadian genes in a dataset. These principal components have a circular relationship and we use $\phi = arctan\left( \dfrac{PC2}{PC1}\right)$ with $\phi \in [-\pi , \pi ]$ rescaled to $\phi \in [0, 24]$h to assign circadian times $\phi$ to all samples in a dataset. If a dataset is a gene expression time series, we randomise its temporal order before we apply the PCA.

### Supplementary Information

Supplementary Information 1.

Supplementary Information 2.

Supplementary Information 3.

Supplementary Information 4.

Supplementary Information 5.

Supplementary Figures.

## Data Availability

We downloaded gene expression counts of four public datasets: Zhang et al.^25^ (mouse, 24 samples, samples were collected every 2 h, ncbi.nlm.nih.gov/geo (GSE54652) at Apr 19 2022) Wolff et al.^26^ (mouse, 12 samples per age (young, aged, old), samples were collected every 4 h, ncbi.nlm.nih.gov/geo (GSE201207) at Nov 10 2022) Mure et al.^27^ (baboon, 12 samples, samples were collected every 2 h, ncbi.nlm.nih.gov/geo (GSE98965) at Apr 22 2022) TCGA-LUAD (human, 51 normal and 58 tumor samples with unrecorded collection times, portal.gdc.cancer.gov at Mar 25 2022) We applied a log2 transformation to the data if it was not already log2 transformed.

## Code availability

The code of this publication is available at https://github.com/saskia-grabe/Quantification-of-circadian-rhythms-in-mammalian-lung-tissue-snapshot-data.
