# Supplementary material for: Quantification of circadian rhythms in mammalian lung tissue snapshot data
Source: Sci Rep. 2024 Jul 14;14:16238. doi: 10.1038/s41598-024-66694-7 (PMC11247089; doi:10.1038/s41598-024-66694-7)
Supplement: Supplementary file 6 — Supplementary Figures. [file 41598_2024_66694_MOESM6_ESM.pdf]

Supplemental information

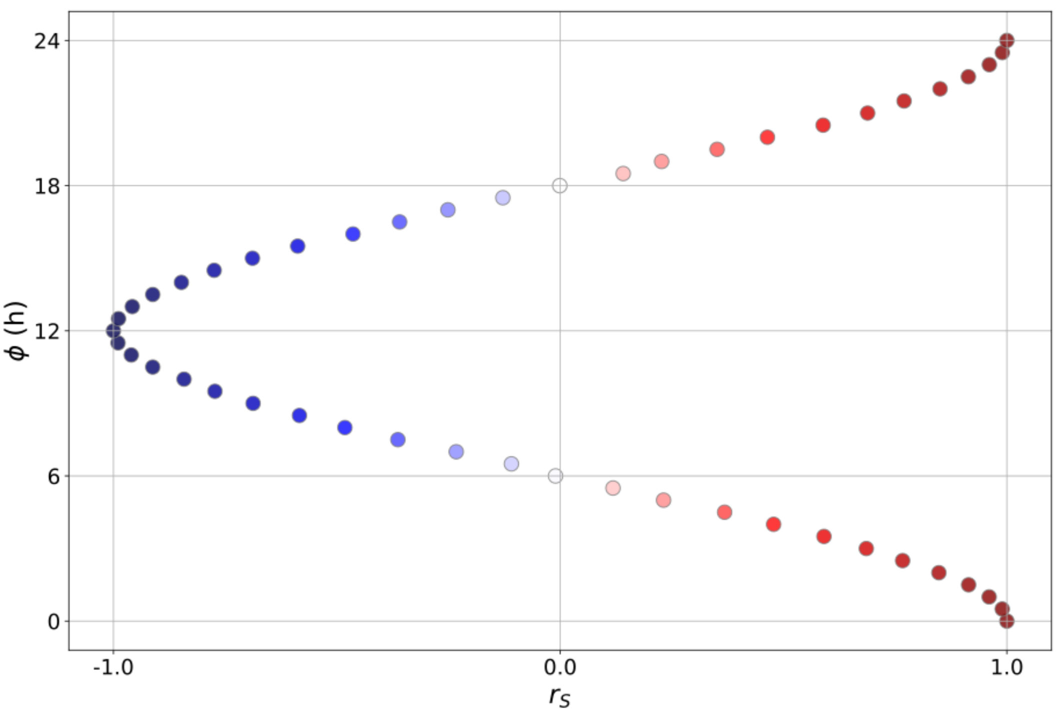

Figure S1. Two sinusoids have a regular relationship between their phase differences and their Spearman rank correlations. Relationship between the Spearman rank correlation r_S and the phase difference between the oscillations of the sine functions $x_1(t) = sin(\frac{2\pi}{24}t)$ and $x_2(t) = sin(\frac{2\pi}{24}t + \phi)$ for varying ϕ. The colors indicate r_S.

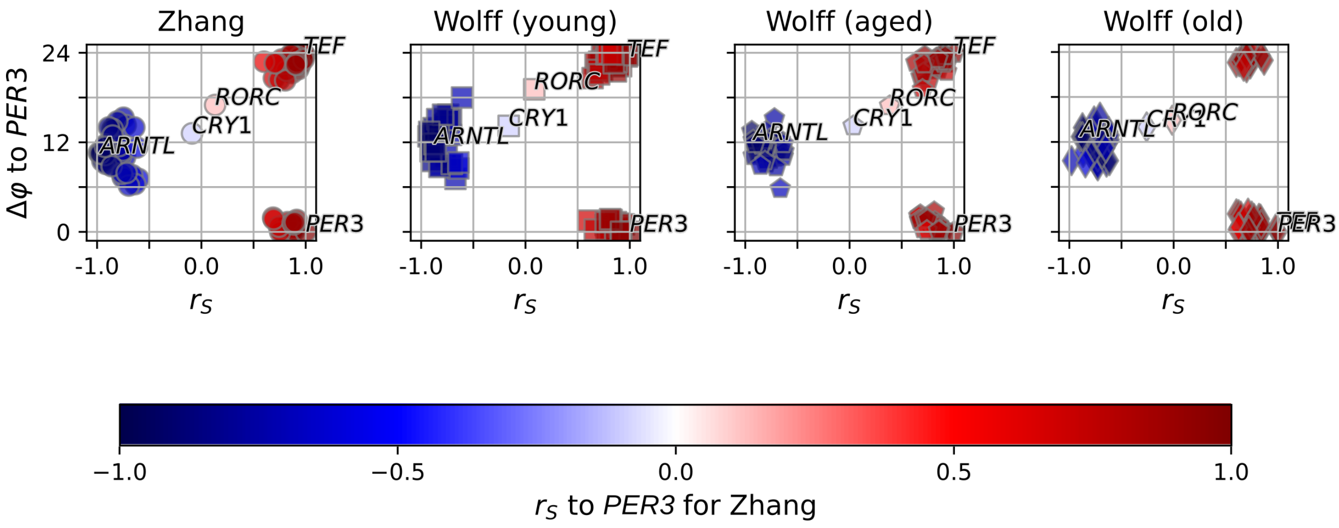

Figure S2. Phase differences between circadian genes selected by Spearman rank correlations are similar across mouse datasets. Relationship between the Spearman rank correlation and the phase difference between the oscillations of *PER3* and 107 murine circadian lung genes for gene expression data of mouse lung of Zhang et al. and Wolff et al.

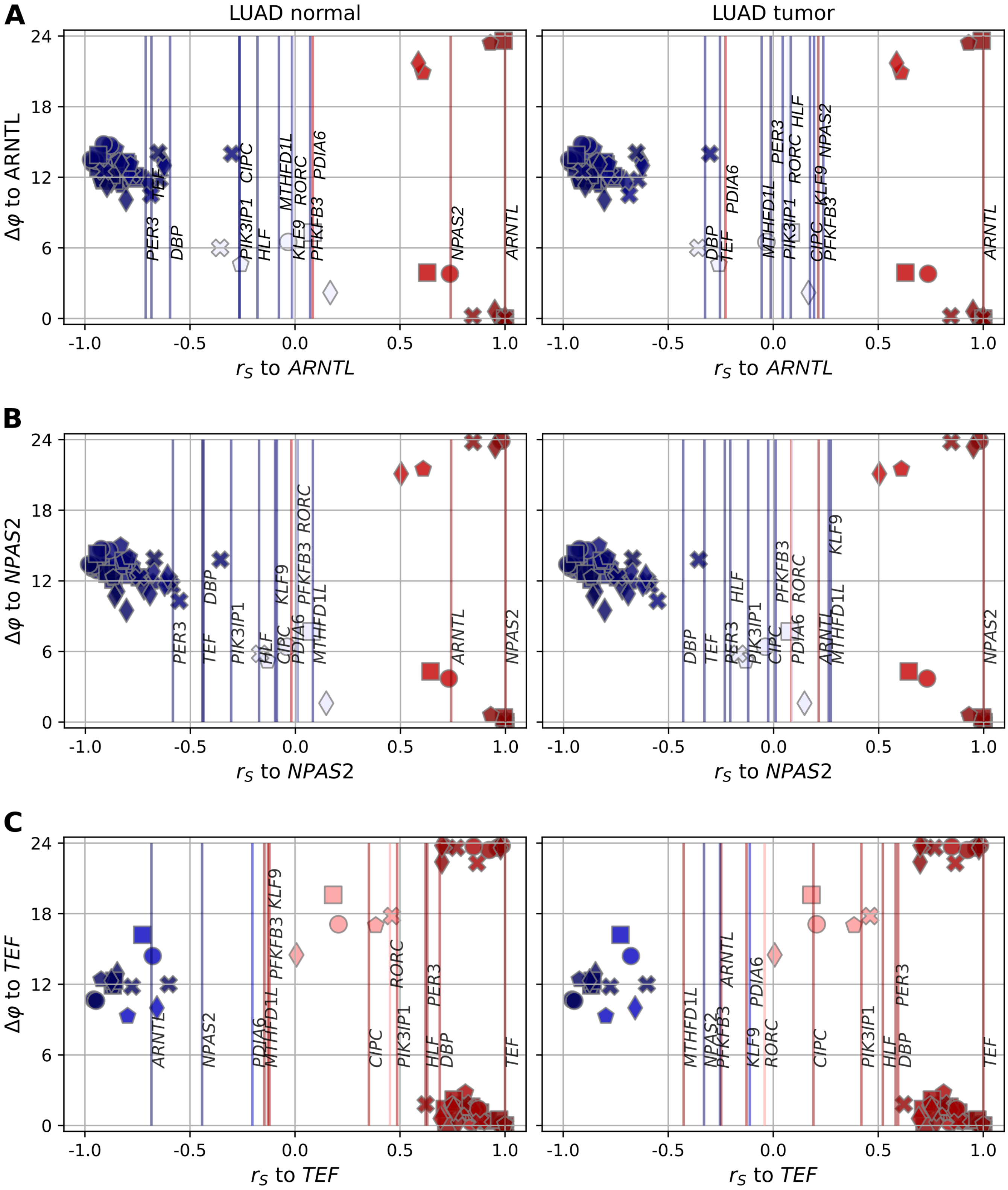

Figure S3. Circadian genes with altered rhythmicity in lung cancer have altered Spearman rank correlations to the other circadian genes. Spearman rank correlations of *ARNTL* (A), *NPAS2* (B), *TEF* (C) and 13 circadian mammalian lung genes for gene expression snapshots of healthy (left) and cancerous (right) human lung tissue from TCGA-LUAD. Mouse lung data (Zhang et al. and Wolff et al., circles, squares, pentagons, diamonds) and baboon lung data (Mure et al., crosses) is shown for comparison. The colors indicate r_S to *ARNTL*/*NPAS2*/*TEF* for the mouse lung data of Zhang et al.

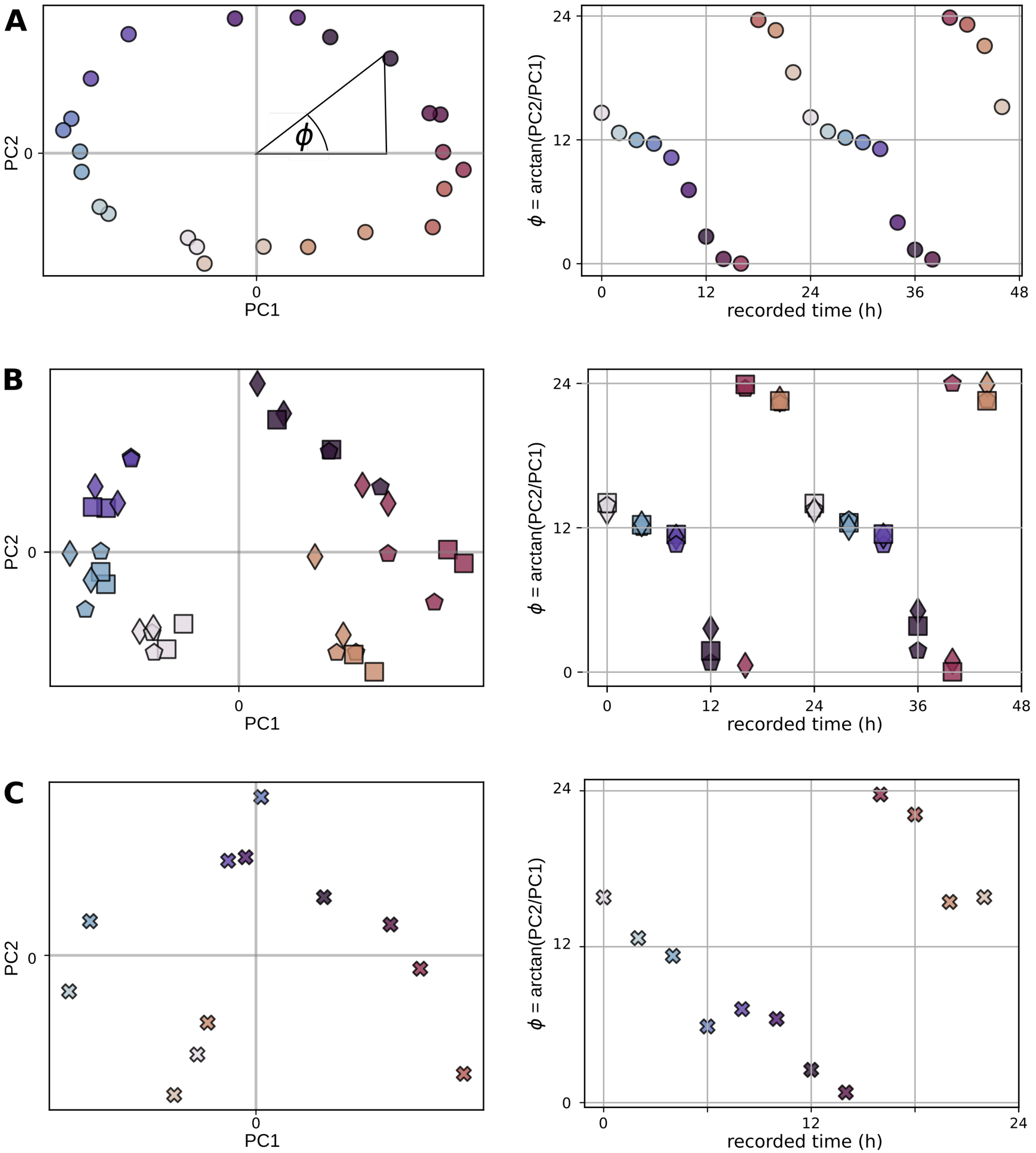

Figure S4. The transcript levels of the shared mammalian circadian genelist contain the information to recover the full circadian day. Circular relationship between PC1 and PC2 after a PCA of the transcript levels of the 13 mammalian circadian genes in mouse lung data from Zhang et al. (A) and Wolff et al. (B), as well as baboon lung data from Mure et al. (C). There is a linear relationship between recorded sample collection times and ϕ. The colors indicate the collection times of the samples.

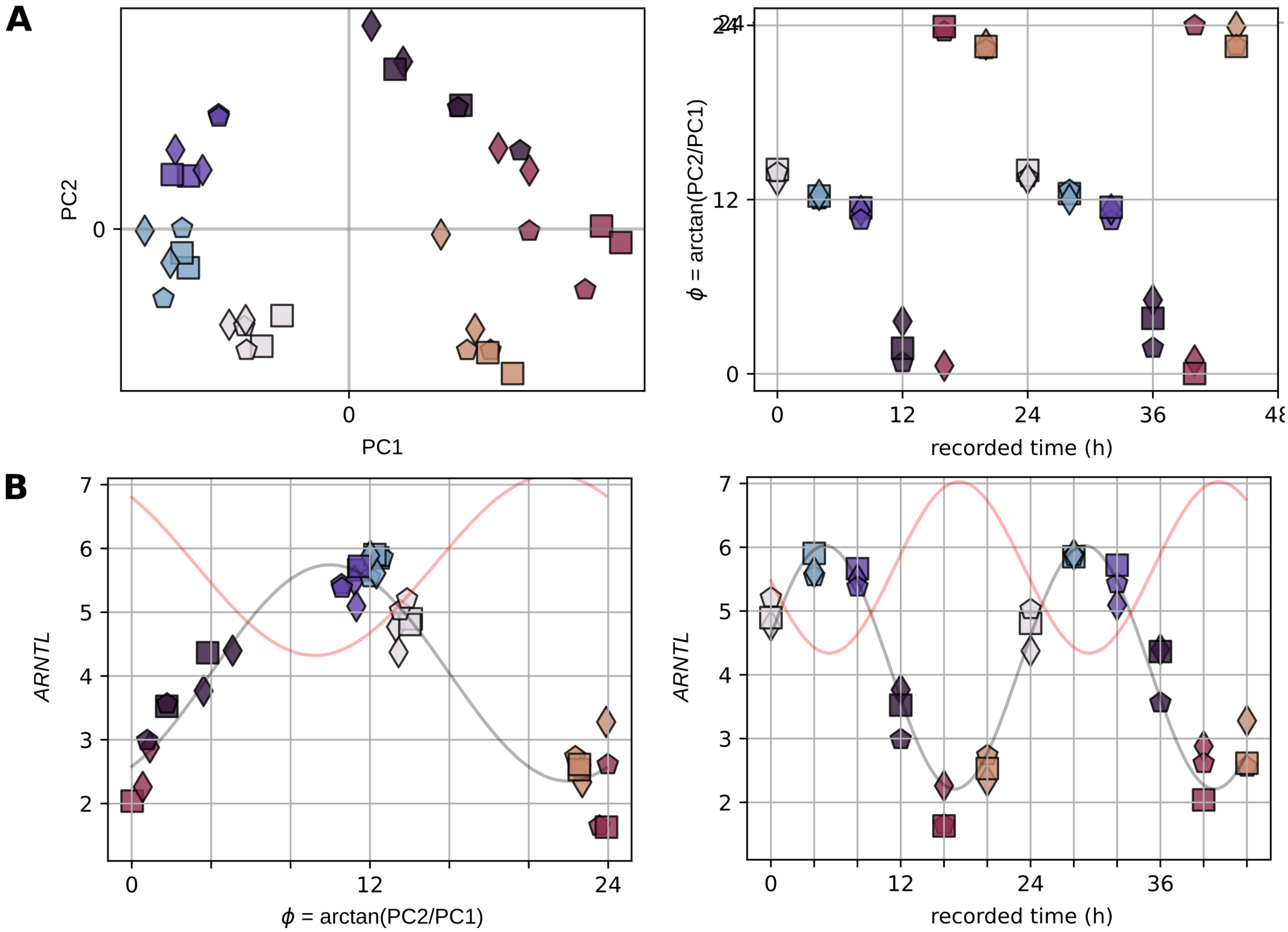

Figure S5. Reconstruction of the circadian transcript level rhythms for a dataset of differently aged mice. (A) Circular relationship between PC1 and PC2 after a PCA of the transcript levels of the 13 mammalian circadian genes in mouse lung data from Wolff et al. All age groups were combined into a single, more heterogeneous dataset before the PCA. (B) Reconstructed and recorded oscillations of *ARNTL* (black line and symbols) and *PER3* (red line). The colors indicate the recorded times of the samples.

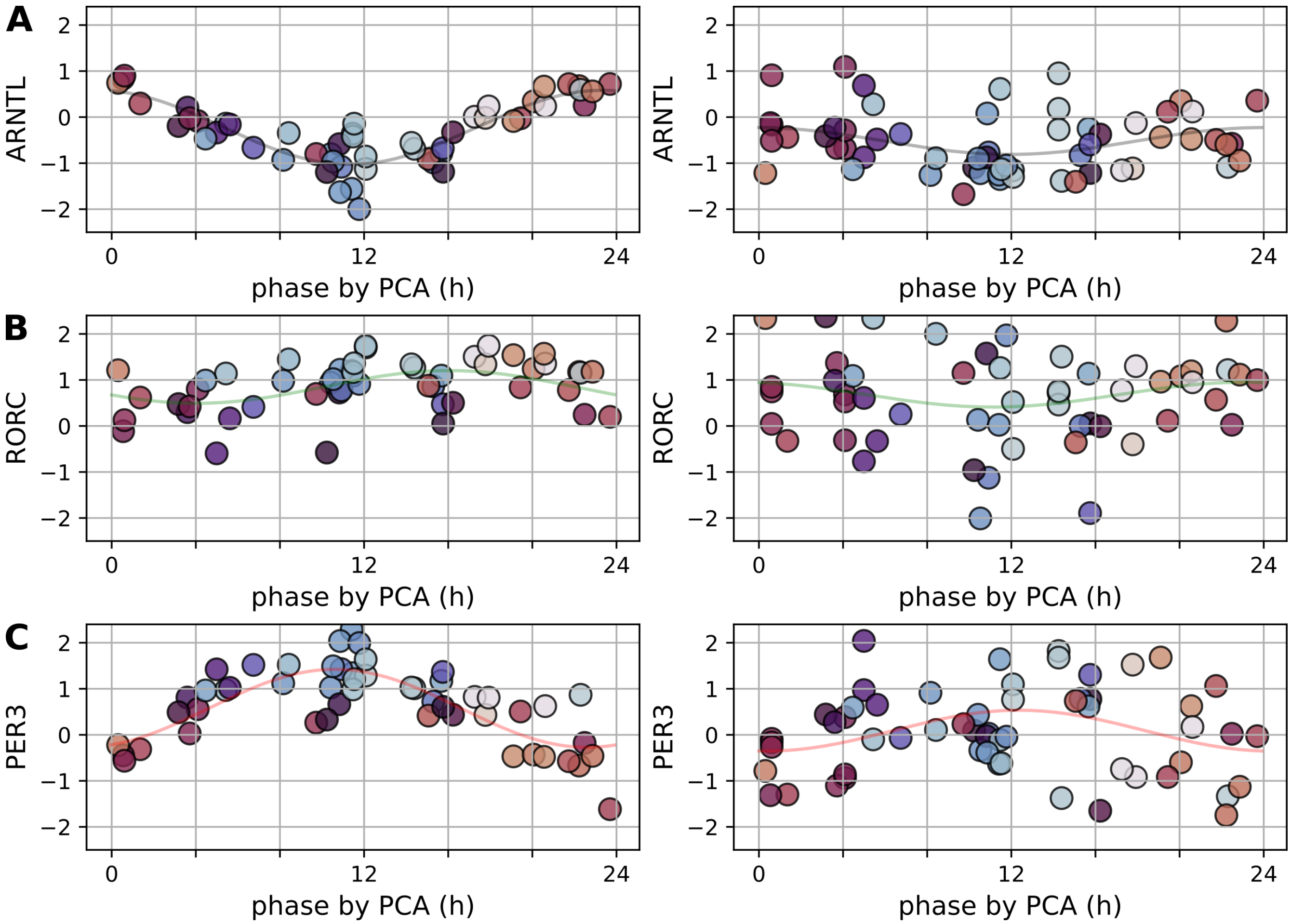

Figure S6. Circadian rhythms of important clock gene transcripts are weak in lung cancer. Reconstructed gene expression time series of *ARNTL* (A), *RORC* (B) and *PER3* (C) in healthy (left) and cancerous (right) human lung tissue. The colors indicate the position of the (matched healthy) samples in gene expression space of *PER3* and *RORC* (Figure 5D in main text).
